# A new Brazilian *Passiflora* leafminer: *Spinivalva gaucha*, gen. n., sp. n. (Lepidoptera, Gracillariidae, Gracillariinae), the first gracillariid without a sap-feeding instar

**DOI:** 10.3897/zookeys.291.4910

**Published:** 2013-04-17

**Authors:** Rosângela Brito, Gislene L. Gonçalves, Hector A. Vargas, Gilson R. P. Moreira

**Affiliations:** 1 PPG Biologia Animal, Departamento de Zoologia, Instituto de Biociências, Universidade Federal do Rio Grande do Sul, Av. Bento Gonçalves 9500, Porto Alegre RS, 91501-970, Brazil; 2 Departamento de Genética, Instituto de Biociências, Universidade Federal do Rio Grande do Sul, Av. Bento Gonçalves, 9500. Porto Alegre, RS 91501-970, Brazil; 3 Departamento de Recursos Ambientales, Facultad de Ciencias Agronómicas, Universidad de Tarapacá, Casilla 6-D, Arica, Chile; 4 Departamento de Zoologia, Instituto de Biociências, Universidade Federal do Rio Grande do Sul, Av. Bento Gonçalves 9500, Porto Alegre RS, 91501-970, Brazil

**Keywords:** Atlantic Rain Forest, gracillariids, leaf-mining moths, Neotropical region, passion vines, Passifloraceae

## Abstract

Male, female, pupa, larva and egg of a new genus and species of Gracillariidae (Gracillariinae), *Spinivalva gaucha* Moreira and Vargas from southern Brazil are described and illustrated with the aid of optical and scanning electron microscopy. A preliminary analysis of mitochondrial DNA sequences including members of related lineages is also provided. The immature stages are associated with *Passiflora actinia*, *Passiflora misera* and *Passiflora suberosa* (Passifloraceae), and build mines on the adaxial leaf surface. Initially the mines are serpentine in shape, but later in larval ontogeny become a blotch type. Although the larvae are hypermetamorphic as in other Gracillariidae, there is no sap-feeding instar in *Spinivalva gaucha*; the larva feeds on the palisade parenchyma, thus producing granular frass during all instars. Pupation occurs outside the mine; prior to pupating, the larva excretes numerous bubbles that are placed in rows on the lateral margins of the cocoon external surface. This is the second genus of gracillariid moth described for the Atlantic Rain Forest, and the second gracillariid species known to be associated with Passifloraceae.

## Introduction

Gracillariidae is a diverse lineage of leaf-mining Lepidoptera, with a total of 102 recognized genera and 1,880 species, distributed worldwide except for Antarctica; 24 of the genera (181 species) have been recorded in the Neotropical region (De Prins and De Prins 2013). Only four genera are recognized as endemic to South America; one occurs in the Atlantic Rain Forest of Brazil (*Leurocephala* Davis & McKay, 2011) and three in Chile: one in the southern Valdivian forests (*Prophyllocnistis* Davis, 1994) and two in the northern coastal valleys of the Atacama Desert (*Angelabella* Vargas & Parra, 2005; *Chileoptilia* Vargas & Landry, 2005) (Davis 1994, Vargas and Landry 2005, Vargas and Parra 2005, Davis et al. 2011). Only 29 gracillariid species have been recorded up to now for the Amazon and Atlantic rain forests of Brazil. This small number likely results from low collecting effort, since microlepidopterans in general have been undercollected in these biomes. Recent surveys conducted in a relatively small area of Central America suggested that a single gracillariid genus (*Phyllocnistis* Zeller, 1848) may include hundreds of species (Davis and Wagner 2011).

Almost all of what is known about the diversity of Brazilian gracillariids is concerned with the adult stage, in general associated with the original species descriptions, which were provided primarily by the pioneer work of Meyrick (1921, 1928, 1932). Several recent studies have suggested that the most informative characters for distinguishing species of some leaf-mining moths including gracillariids might be found in the pupal morphology (*e.g*., Patočka 1989, Fujihara et al. 2001, Kawahara et al. 2009, Kobayashi et al. 2011). However, studies that include the description of immatures are still in their infancy for microlepidopterans in general, in both the Amazon and Atlantic regions of Brazil (*e.g*., Brown et al. 2004, Becker and Adamski 2008, Brito et al. 2012, Moreira et al. 2012), and thus should be taken as a priority in research on this group.

The Atlantic Rain Forest, where only six species of gracillariids have been recorded up to now (Davis and Miller 1984, Davis et al. 2011, Brito et al. 2012), originally extended for more than 3,300 km along the eastern Brazilian coast and covered more than 1.1 million km^2^ (for a general description, see Morellato and Haddad 2000, Oliveira-Filho and Fontes 2000). Although now restricted to less than 8% of its earlier range, this biome is still among the areas with the greatest diversity of plants and animals on earth, and has long been recognized as extremely rich in endemics (Myers et al. 2000, Carnaval et al. 2009), including Lepidoptera (Freitas et al. 2011). For example, Stehmann et al. (2009) listed 14,552 species of vascular plants for the entire Atlantic Rain Forest, of which 6,933 (49%) are endemic. Considering the wide range of host plants used and the high level of host specificity usually found for the leaf-mining gracillariids in general (Davis 1987), it seems reasonable to predict that hundreds of gracillariid species await description in this understudied, species-rich biome, to which probably most of them are also endemic.

In the course of an ongoing survey on the diversity of microlepidopterans in the Atlantic Rain Forest in southern Brazil, we recently found a leaf-mining gracillariid associated with Passifloraceae. A search of the literature indicated that this taxon is distinct from other described genera of Gracillariidae, and therefore a new genus is proposed herein. We describe and illustrate all the life stages of this new species, and provide a preliminary characterization of its life history, including histological aspects of the leaf mine. We also present a preliminary analysis of mitochondrial DNA sequences, including members of related genera.

## Materials and methods

Specimens used in the study were reared in small plastic vials under controlled abiotic conditions (14 h light / 10 h dark; 25 ± 2 °C) in the Laboratório de Morfologia e Comportamento de Insetos, Departamento de Zoologia, Universidade Federal do Rio Grande do Sul (UFRGS), Porto Alegre city, Rio Grande do Sul State (RS), Brazil, from May 2011 through December 2012. They came from field-collected leaves with eggs, mines with feeding larvae inside, and pupae on plant shoots of *Passiflora actinia* Hook. (São Francisco de Paula municipality, RS), *Passiflora misera* Kunth and *Passiflora suberosa* L. (Porto Alegre municipality, RS).

Immature stages were fixed in Dietrich´s fluid and preserved in 75% ethanol. For descriptions of the gross morphology, the specimens were cleared in a 10% potassium hydroxide (KOH) solution and slide-mounted in either glycerin jelly or Canada balsam. Observations were performed with the aid of a Leica® M125 stereomicroscope. Measurements were performed with the aid of an ocular micrometer (precision = 0.01mm). Structures selected to be drawn were previously photographed with a Sony^®^ Cyber-shot DSC-H10 digital camera mounted on the stereomicroscope. Vectorized line drawings were then made with the software Corel Photo-Paint^®^ X3, using the corresponding digitalized images as a guide. At least five specimens were used for the descriptions of each life stage or instar.

For scanning electron microscope analyses, additional specimens were dehydrated in a Bal-tec® CPD030 critical-point dryer, mounted with double-sided tape on metal stubs, and coated with gold in a Bal-tec® SCD050 sputter coater. They were examined and photographed in a JEOL® JSM5800 scanning electron microscope at the Centro de Microscopia Eletrônica (CME) of UFRGS.

Descriptions of plant anatomy were based on diaphanized, field-collected leaf-mines (n =5) from *Passiflora actinia* shoots that were fixed in FAA (37% formaldehyde, glacial acetic acid, and 50% ethanol, 1:1:18, v/v), stained with rose bengal (aqueous solution: 200 mg/1) and mounted either whole or in freehand section in glycerin on slides, following a procedure described in detail by Brito et al. (2012).

### Molecular analysis

High-quality DNA was purified from larval tissue using the organic method of Cetyl Trimethyl Ammonium Bromide (CTAB) to investigate (i) levels of genetic variation within *Spinivalva* specimens collected in different localities and from different host plants (*Passiflora misera*, *Passiflora suberosa* and *Passiflora actinia*) and (ii) reconstruct phylogenetic relationships of this new genus among and within the *Parectopa* group of gracillariids. A total of nine field-collected specimens from three populations: 1) Porto Alegre, RS, from *Passiflora suberosa* and *Passiflora misera* (Pop. 1); 2) São Francisco de Paula, RS, from *Passiflora actinia* (Pop. 2) and 3) Curitiba, PR, also from *Passiflora actinia* (Pop. 3). They were used to amplify 1.5 kb of mitochondrial genes cytochrome *c* oxidase subunit I (CO-I), transfer RNA (tRNA-Leu), and cytochrome *c* oxidase subunit II (CO-II). For the PCR amplification we used the primer pairs Jerry + Pat II for the first segment (700 bp), and Patrick + Eva for the second (800 bp), following the procedure described by Caterino and Sperling (1999). Additionally, we amplified genetic material from three specimens of *Spinivalva*, using the universal barcode primers LCO1490 (5’-ggtcaacaaatcataaagatattgg-3’) and HCO2198 (5’-taaacttcagggtgaccaaaaaatca-3’), following the procedure of Folmer et al. (1994). We obtained variants that exactly matched the region previously sequenced in 6 representative taxa of the *Parectopa* group of gracillariids, downloaded from GenBank and incorporated into our analysis (Table 1). The remaining PCR products were treated with exonuclease I and shrimp alkaline phosphatase (ExoSAP) (Fermentas Inc.), sequenced using the BigDye sequencing kit and analyzed in an ABI 3730XL DNA Analyzer (Applied Biosystems Inc.). Sequences were aligned and visually inspected using the algorithm Clustal X in MEGA 5 (Tamura et al. 2011) running in full mode with no manual adjustment. The dataset of 1.5 kb generated for specimens of *Spinivalva* from three different localities was deposited in GenBank and BOLD, under the accession numbers KC512114 to 512123 and GRABR001-13 to 010-13, respectively. The phylogenetic tree was reconstructed based on Bayesian inference and implemented in BEAST 2.0 (Drummond et al. 2012) to recover (i) the evolutionary distance within *Spinivalva* taxa from different localities and host plants, and (ii) relationships of *Spinivalva* among the lineages of gracillariids surveyed in this study. In both trees, the HKY85 model of sequence evolution (Hasegawa et al. 1985) was used with empirical base frequencies and 4 gamma categories. A relaxed uncorrelated log-normal clock was used, with no fixed mean substitution rate and a Yule prior on branching rates. We used four independent runs of 10 million generations, with the first 500,000 of each run discarded as burn-in. Posterior probabilities were used as an estimate of branch support. The species-level tree was unrooted, while the genus-level was rooted with a species of Bucculatricidae (*Bucculatrix ulmella* Zeller, 1848).

**Table 1.** Specimens used to investigate phylogenetic relationships of *Spinivalva* within the *Parectopa* group of gracillariids (following Kawahara et al. 2011). See Material and Methods for a detailed description of *Spinivalva* specimens.<br/>

**Taxa**	**Voucher**	**GenBank Accession Number**
**Ingroup**		
GRACILLARIIDAE		
*Conopomorpha sinensis* Bradley, 1986	-	HQ824810
*Epicephala mirivalvata* Li, Wang & Zhang, 2012	-	JX231168
*Leurocephala schinusae* Davis & McKay, 2011	RDOPO385-10	HM382093
*Liocrobyla desmodiella* Kuroko, 1982	G95AK	GU816416
*Parectopa* sp.	10-JDWBC-0213	HM863870
*Spinalva gaucha* sp. n.	LMCI 186-12	KC512112
	LMCI 164-15	KC512113
*Spinivalva* sp. 1	LMCI 169-A1	KC512114
*Stomphastis* sp.	USNM:ENT 00455002	JF415895
**Outgroup**		
BUCCULATRICIDAE		
*Bucculatrix ulmella* Zeller, 1848	RMNH.INS.18466	JX215365

### Museum collections

Abbreviations of the institutions from which specimens were examined are:

**DZUP** Coll. Padre Jesus S. Moure, Departamento de Zoologia, Universidade Federal do Paraná, Curitiba, Paraná, Brazil.

**LMCI** Laboratório de Morfologia e Comportamento de Insetos, Universidade Federal do Rio Grande do Sul, Porto Alegre, Rio Grande do Sul, Brazil.

**MCNZ **Museu de Ciências Naturais, Fundação Zoobotânica do Rio Grande do Sul, Porto Alegre, Rio Grande do Sul, Brazil.

**MCTP** Museu de Ciências e Tecnologia da Pontifícia Universidade Católica do Rio Grande do Sul, Porto Alegre, Rio Grande do Sul, Brazil.

## Results

### Spinivalva

Moreira & Vargas gen. n.

urn:lsid:zoobank.org:act:F4A4BC02-04D7-41C8-9328-B477EA4E5592

http://species-id.net/wiki/Spinivalva

Figs 1 - 11

#### Type species.

*Spinivalva gaucha* Moreira and Vargas, sp. n\. by present designation.

#### Diagnosis.

*Spinivalva* males show several abdominal and genital features that in conjunction differentiate this taxon from all known gracillariid genera: 1) saccular extension of valva abruptly narrowing distally, forming a single, medially bent process bearing a stout sensillum at the apex; 2) aedeagus tubular, slender, straight and long, ending as a sharply pointed spine; 3) saccus with anterior process long and tubular; 4) two pairs of coremata, each with two unit types that are formed by an external hair pencil and a tubular, membranous, corrugated pouch. In the female genitalia, the circular ostium bursae is located near the anterior margin of sternum VII, having a membranous corpus bursae associated with an accessory bursa, with no signum. The larvae construct mines on the adaxial surface of passion-vine leaves; initially the mines are serpentine in shape but later in ontogeny become a blotch type. Unlike all known stages of other leaf-miner gracillariids, *Spinivalva gaucha* has no larval sap-feeding instars; all instars of its larvae have a conspicuous spinneret and mandibles of the chewing type, and feed on the palisade parenchyma after hatching. Pupation occurs outside the mine; the larva excretes numerous bubbles that are aligned on the lateral margins of the cocoon surface prior to pupation.

#### Description.

**Figure 1.**
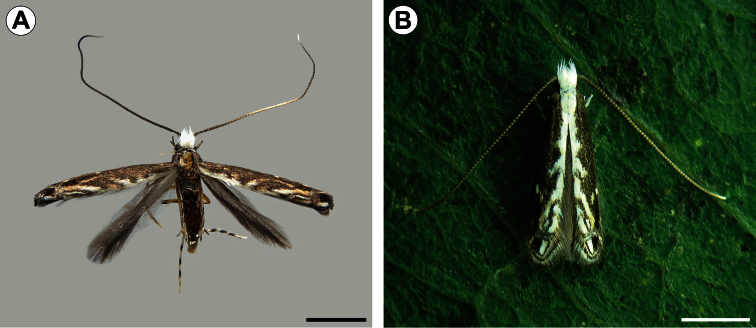
*Spinivalva gaucha* adult, dorsal view: **A** wings spread, pinned, dorsal view **B** wings folded, on *Passiflora actinia* leaf. Scale bars = 1.0 mm.

**Figure 2.**
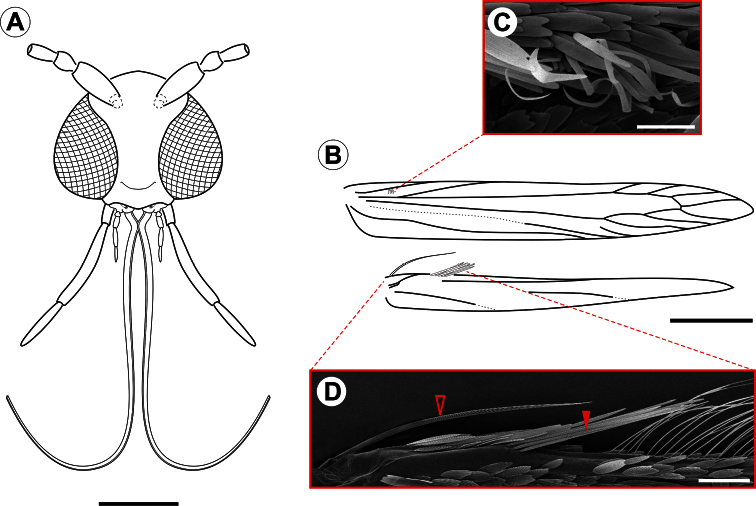
*Spinivalva gaucha* adult morphology: **A** head, anterior view **B** fore- and hind-wing venation **C** detail of retinaculum **D** detail of basal frenulum (open arrow) and more distal pseudofrenulum (closed arrow). Scale bars = 0.2, 0.5 mm; 50, 100 µm, respectively.

**Figure 3.**
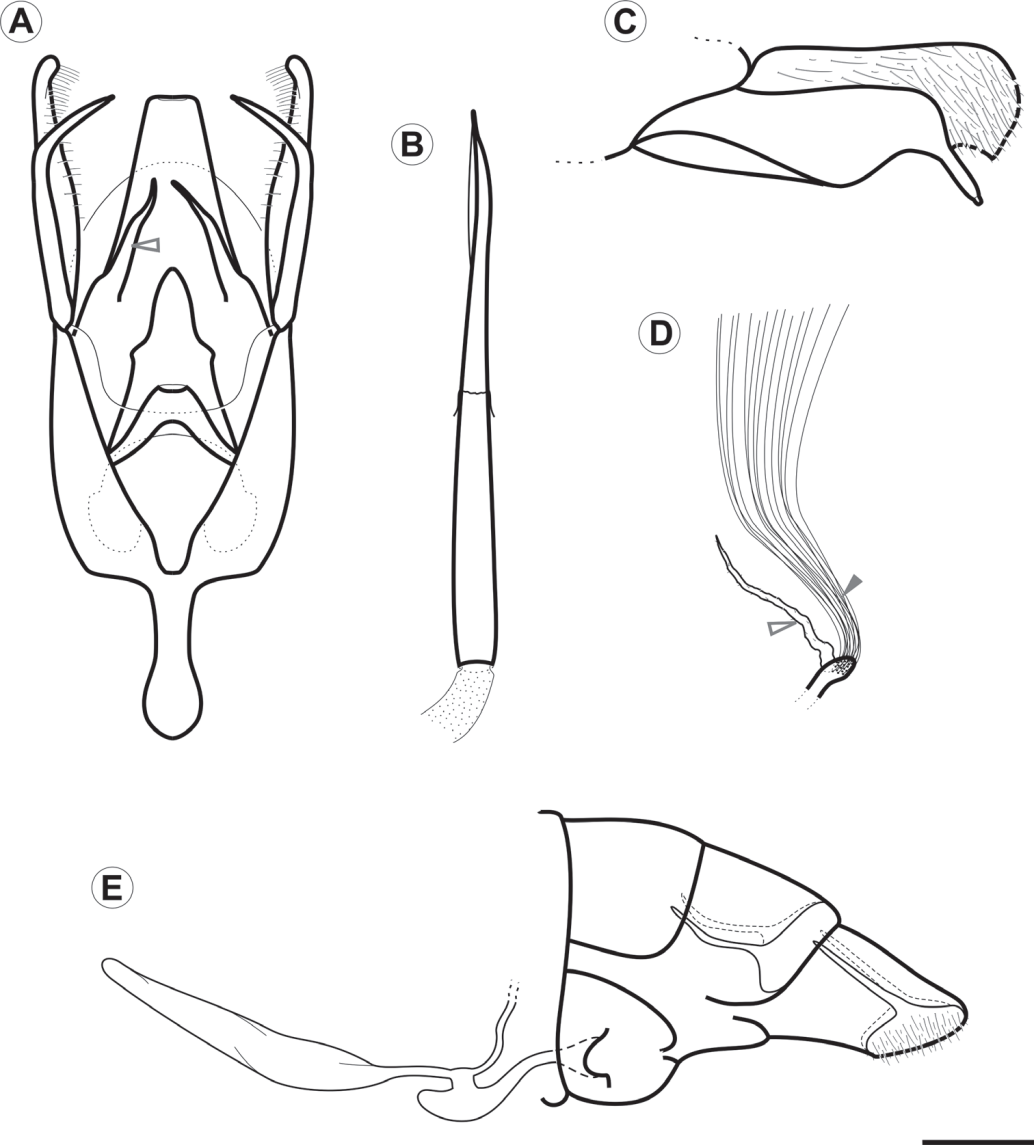
Genital morphology of *Spinivalva gaucha* under light microscopy:**A** male genitalia, ventral view (aedeagus omitted; open arrow indicates gnathal lobe) **B** aedeagus, lateral view **C** male right valve, median view **D** units of the coremata anterior pair, ventral view (open and closed arrows indicate tubular pouch and hair pencil, respectively) **E** female genitalia, lateral view. Scale bar = 0.2 mm.

**Figure 4.**
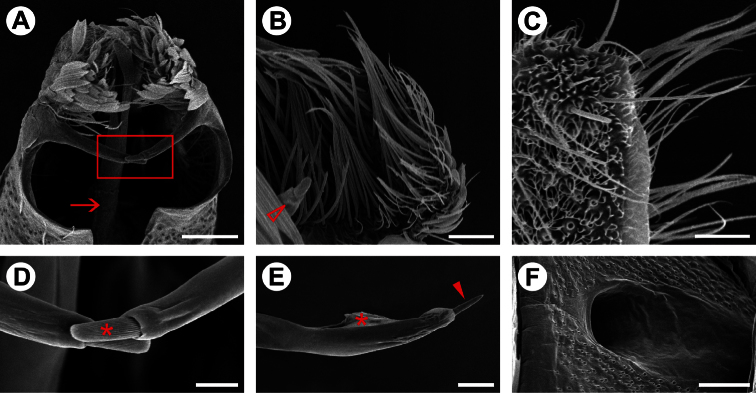
Genital morphology of *Spinivalva gaucha* under scanning electron microscopy:**A** male valvae (scales partly removed), showing saccular processes in crossed position and aedeagus (indicated by arrow), ventral view **B** setae of costa valvular in detail (open arrow indicates distal portion of saccular process), median view **C** female papilla annalis in detail, latero-dorsal view **D** saccular processes in detail (squared area in **A**; asterisk indicates distal sensillum of the right process) **E** caudal portion of aedeagus, showing terminal spine (indicated by closed arrow) and vesica (indicated by asterisk), lateral view **F** female ostium bursae, ventral view. Scale bars = 50, 25, 20, 10, 25, 50 µm, respectively.

**Adult** (Figs 1–4). Male and female similar in size and color. Small moth, forewing length 2.78–3.61 mm (n = 5). *Head* (Fig. 2A): Vestiture moderately smooth, with a large, light-gray dorsal scale tuft that curves forward to the frons; scales slender, with apices slightly rounded. Eye relatively large, rounded, with dorsal margin slightly concave; vertical diameter ~ double minimum interocular distance across frons (n = 6). Antenna filiform, long, exceeding length of forewing; scape slightly elongate, ~ 2.4× length of pedicel; flagellomeres completely encircled by single, dense row of slender scales. Labrum trilobed, pilifers well developed, triangular. Mandible absent. Haustellum naked, elongate, ~ 2.0× length of labial palpus. Maxillary palpus short, smoothly scaled, 4-segmented; ratios of segments from base: ~1.0 : 2.2 : 3.6 : 3.5. Labial palpus smoothly scaled, moderately long, bent anteriorly and upward; ratio of segments from base: ~1.0 : 4.6 : 0.3. *Thorax*:Forewing (Fig. 2B) lanceolate, with 12 veins, all arising separately from the cell and reaching the margin; L/W index ~ 7.3; retinaculum consisting of few subcostal, narrow, flat, longer, loosely coiled scales (Fig. 2C); discal cell ~ 0.8× length of forewing (n = 4) ending near distal fifth of wing margin; R5-branched; R1 ending near proximal third of wing margin; M3-branched, CuA not branched, and faded basally; CuP weak proximally and not stalked, with 1A+2A that is well developed, extending past midlength of posterior margin. Hindwing (Fig. 2B) extremely lanceolate, L/W index ~ 9.6, ~ 1/8 forewing in length; male frenulum (Fig. 2D) a single stout bristle; female with frenulum divided at base, then fused for nearly its entire length and appearing as a single stout bristle; pseudofrenulum consisting of ~8 modified scales arising in two to three irregular rows near Sc+R_1_ ending at circa 1/5 anterior margin; Rs faded proximally, ending at circa 1/3 anterior margin; M and CuA unbranched, both faded proximally and weak distally, ending at circa 1/3 and 2/3 of posterior margin. Legs with tibial spur pattern 0-2-4; epiphysis present. Tibial length ratios (anterior / middle / posterior legs) ~ 0.55/0.85/1.0. *Abdomen*: Male with segments VII-VIII complex and reduced, except for enlarged tergum VIII; segment VII reduced to narrow, almost completely sclerotized ring; tergum VIII elongate, hoodlike, partly covering tegumen; sternum VII bearing two pairs of coremata, arising from distal apex of rodlike sclerites that protrude from intersegmentary membrane VII-VIII; each coremata (Fig. 3D) bearing two types of units – an external hair pencil ( ~ valva in length) and a tubular, membranous, corrugated pouch; pouches of anterior pair ~ ½ hair pencil in length; those of caudal pair double in size (near to hair pencil in length). Female postabdominal segments unmodified.

**Male genitalia** (Figs 3A–C, 4A, B, D, E). Uncus absent. Tegumen broad, hood-shaped, mostly membranous, with shallow apical notch. Pair of long, distally narrower, membranous lobes arising ventrally beneath tegumen. Vinculum long, broadly V-shaped, extending laterally along base of valva. Saccus well developed, U-shaped; anterior process long and tubiform, ~1/2 length of valva, apex slightly capitate. Transtilla an arched, sclerotized plate joining bases of valvae. Juxta small, a dorsally concave, membranous plate, attached to middle of aedeagus. Aedeagus (Figs 3B, 4E) tubiform, slender, straight and long (~2× valve length), slightly dilated caudally, with subapical, dorsally located concave aperture and ending as sharply pointed spine; entry of ductus ejaculatorius located at anterior end; vesica without cornuti. Valva (Figs 3C, 4A, B, D) broad at base, and deeply divided; costal margin relatively straight and distally rounded; cucullus densely covered by long piliform setae; sacculus with broad lobe abruptly narrowing distally, ending as a medially bent process with apex bearing a stout, blunt sensillum.

**Female genitalia** (Figs 3E, 4C, F). Sternum VII subtriangular; anterior margin linear; posterior margin with narrow notch. Tergum VIII subtriangular. Anterior apophysis with arms slightly curved, similar in length to posterior apophyses. Anal papillae connected dorsally, covered with long piliform setae and microtrichia. Ostium bursae moderately wide, located on anterior margin of sternum VII. Ductus bursa membranous, wider in middle, forming an accessory bursa ~ 1/3 length of corpus bursae. Corpus bursae membranous, gradually broadening posteriorly, ~ twice length of ductus bursae. Ductus seminalis membranous, narrow, inserted on distal portion of accessory bursa. Signum absent.

**Figure 5.**
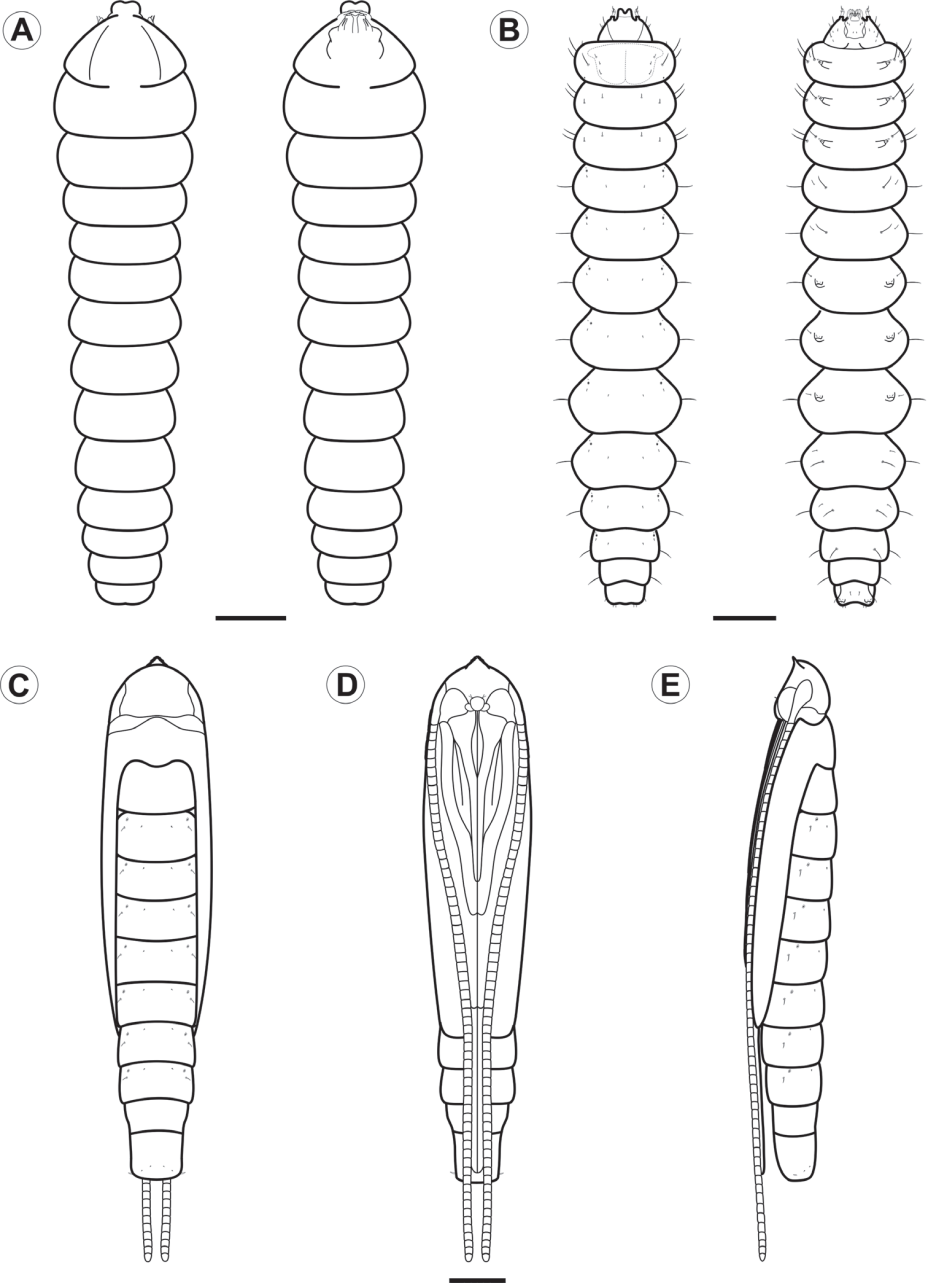
Larval and pupal morphology of *Spinivalva gaucha* under light microscopy: **A** first larval instar, dorsal and ventral views **B** fifth larval instar, dorsal and ventral views **C–E** pupa, dorsal, ventral and lateral views, respectively. Scale bars = 50 µm; 0.5, 0.5 mm, respectively.

**Figure 6.**
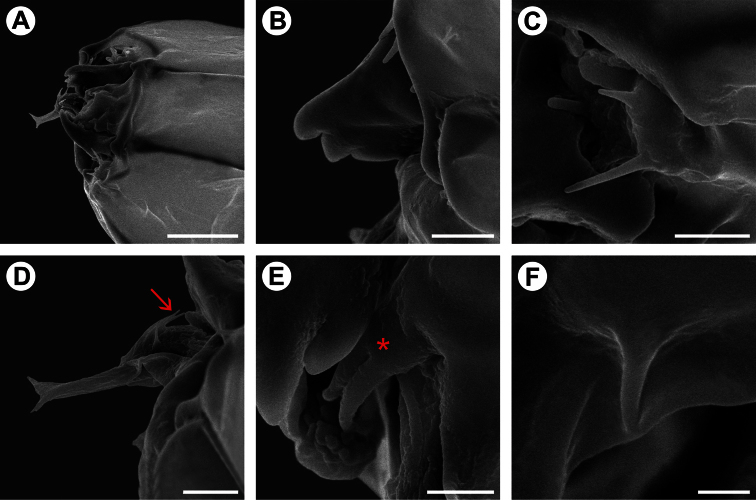
Scanning electron micrographs of *Spinivalva gaucha* first larval instar: **A** head, general, dorso-lateral view **B** mandible, dorsal view **C** antenna, lateral view **D** spinneret, lateral view; seta indicates hypopharyngeal papillae **E** maxilla (asterisk), lateral view **F** labial palpi. Scale bars = 20, 5, 5, 5, 3, 1 µm, respectively.

**Figure 7.**
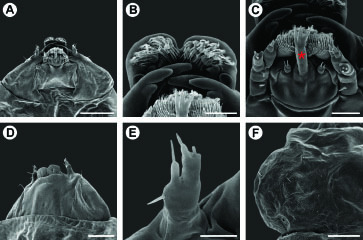
Scanning electron micrographs of *Spinivalva gaucha* third larval instar: **A** head, general, ventral view **B** labrum, ventral view **C** labium, ventral view (asterisk indicates the spinneret) **D** head, general, dorso-lateral view **E** antenna, antero-ventral view **F** left side of prothoracic shield, dorsal view. Scale bars = 75, 15, 15, 50, 10, 75 µm, respectively.

**Figure 8.**
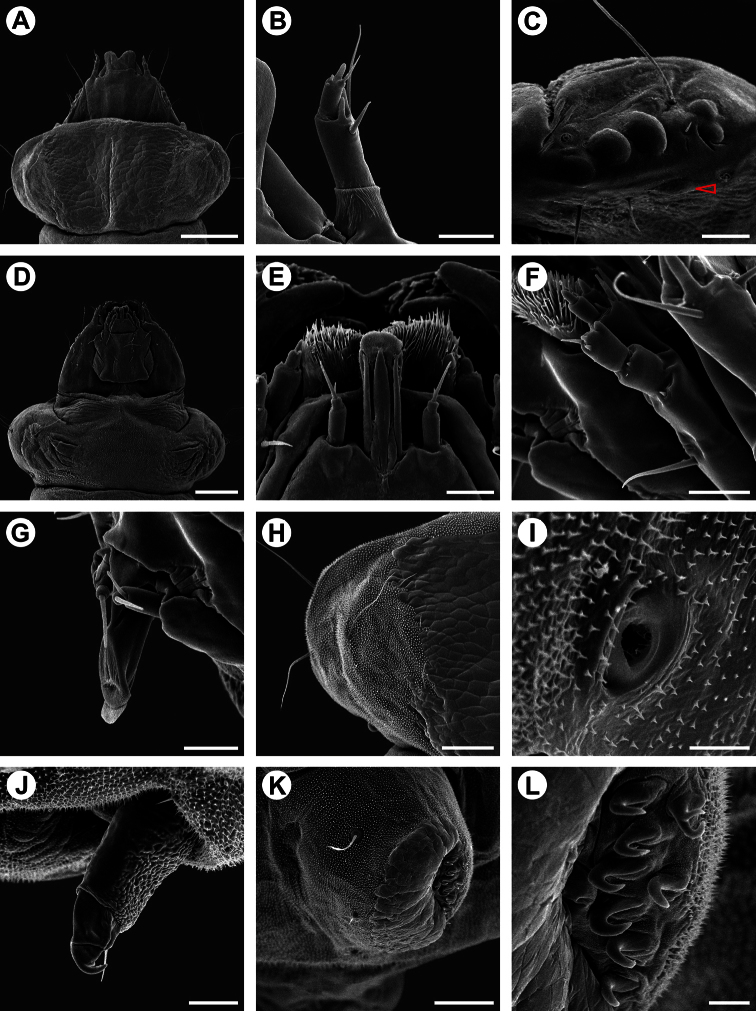
Scanning electron micrographs of *Spinivalva gaucha* fifth larval instar: **A** head and prothorax, general, dorsal view **B** antenna, dorsal view **C** stemmata (open arrow indicates sixth stemma), lateral view **D** head and prothorax, general, ventral view **E** labium, ventral view **F** maxilla, lateral view **G** spinneret, lateral view **H** left side of prothoracic shield, dorsal view **I** prothoracic spiracle, lateral view **J** prothoracic leg, postero-lateral view **K** pseudopodium on A4, antero-lateral view **L** crochets of pseudopodium A4 in detail. Scale bars = 200, 25, 25, 200, 20, 20, 20, 75, 10, 25, 75, 10 µm, respectively.

**Figure 9.**
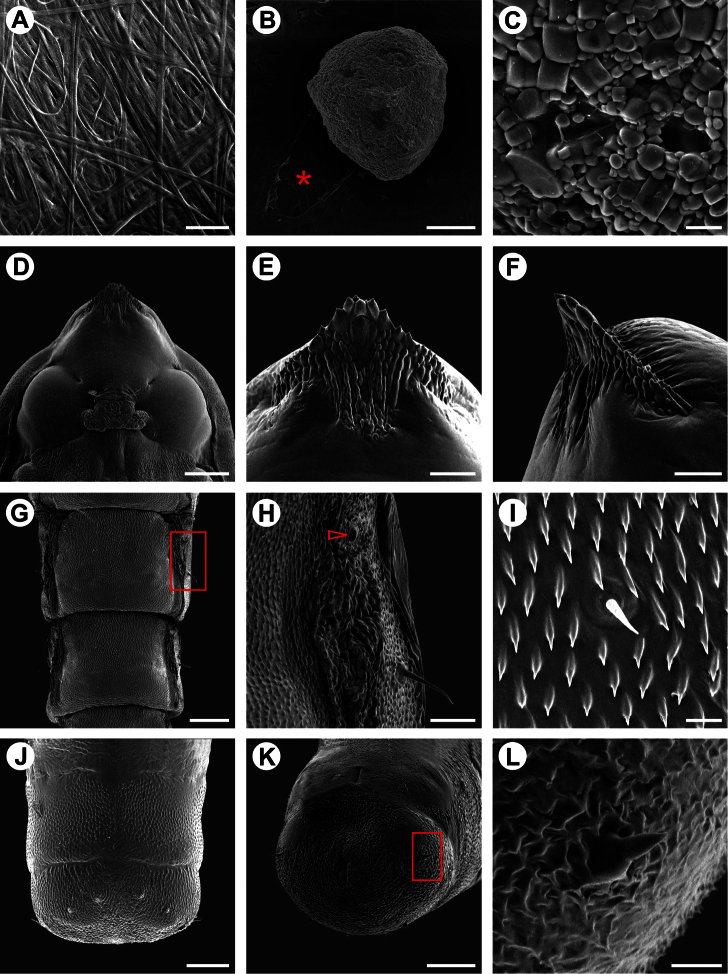
Scanning electron micrographs of *Spinivalva gaucha* pupalcocoon and pupa: **A** weaving pattern of the pupal cocoon upper surface **B** ornamental bubble (asterisk indicates a covered slit, used by the larva to attach the bubble on outside of the cocoon surface) **C** bubble surface in detail **D** head, ventral view **E–F** cocoon-cutter, ventral and lateral views, respectively **G** abdominal segments, dorsal view **H** abdominal segment A3 (detail of area marked with a rectangle in **G**; open arrow indicates spiracle) **I** setae and microtrichia occurring on central portion of A1-A7 **J–K** last abdominal segments, dorsal and ventro-posterior views, respectively **L** spine of last abdominal segment (detail of squared area marked in **K**). Scale bars = 20, 75, 5, 150, 50, 50, 200, 50, 10, 75, 75, 10 µm, respectively.

**Figure 10.**
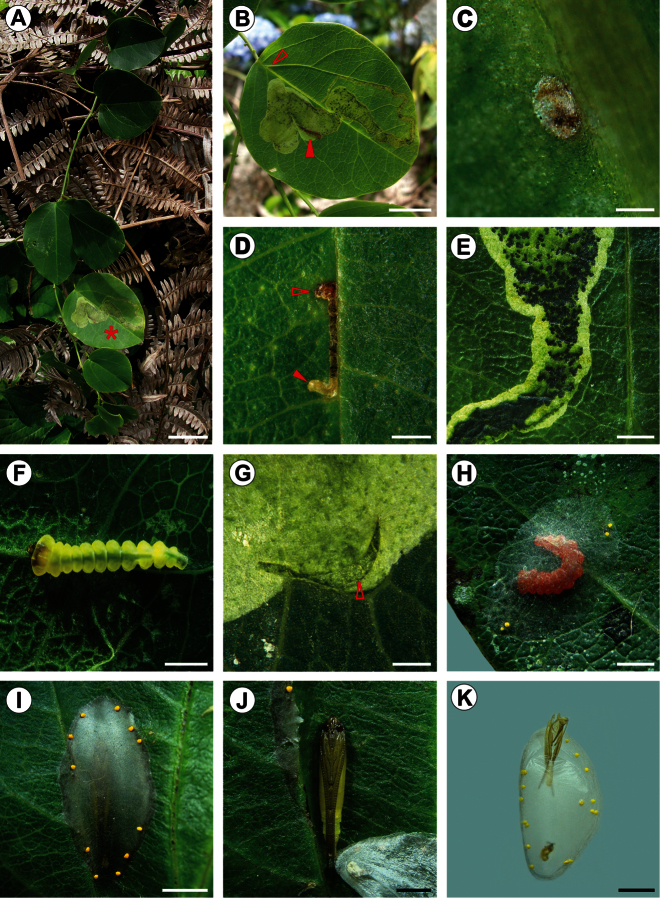
Life history of *Spinivalva gaucha*: **A** *Passiflora actnia* shoot at the type locality **B** *Spinivalva gaucha* mine on upper leaf surface (leaf marked with an asterisk in **A**; open and closed arrows indicate respectively the beginning of a mine and a last-instar larva visible through transparent blotch section of the mine); **C** chorion of empty egg on lower surface **D** first-instar larva (indicated by closed arrow) visible through transparent serpentine section of a young mine (open arrow indicates the beginning of the mine on the upper leaf surface) **E** initial portion of blotch section in detail, showing frass and damage to leaf parenchyma left by last-instar larva within the mine **F** fourth-instar larva in the mine **G** exit hole (arrow) used by a last-instar larva to leave the mine **H** last-instar larva after changing color, building the cocoon outside the mine on the leaf surface **I** cocoon, with pupa visible through transparency **J** pupa in detail, after removing the cocoon **K** pupal exuvium protruding from the cocoon exit hole onto plastic substrate, just after the adult emergence. Scale bars = 20, 10, 0.2, 1.5, 1, 0.5, 0.5, 2, 2, 1, 2 mm, respectively.

**Figure 11.**
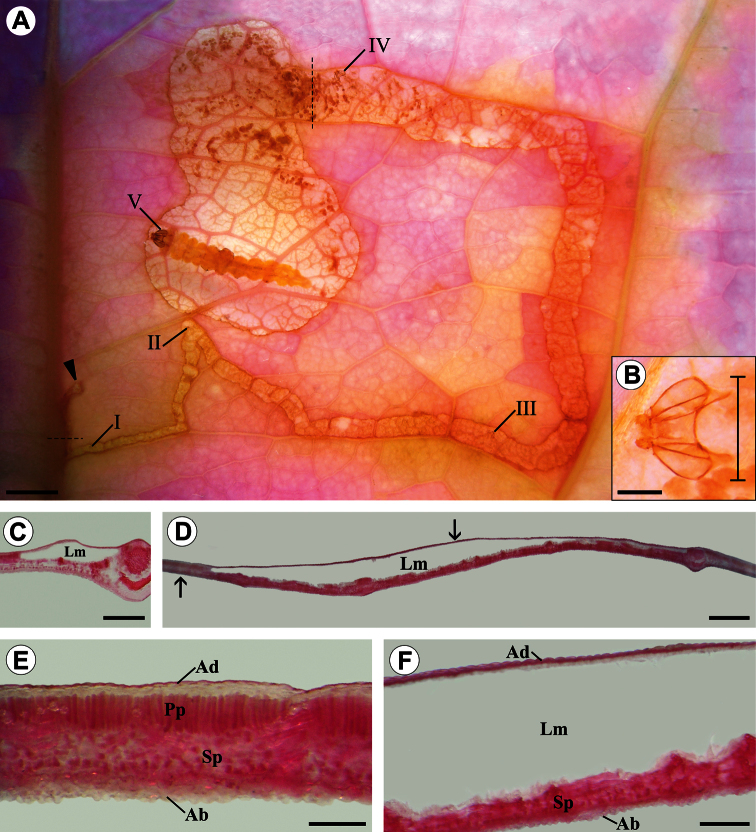
Diaphanized portion and histological sections of a *Passiflora actinia* leaf, showing through transparency the organization levels ofa *Spinivalva gaucha* mine in relation to larval ontogeny: **A** general aspect of the mine, containing a last-instar larva; Roman numerals indicate larval instar numbers and corresponding positions of head capsules in the mine; closed arrow indicates the beginning of the mine **B** detail of head capsule shed by the fourth-instar larva (bar indicates position used for measurement of head-capsule width) **C** transverse section of serpentine portion of the mine (location indicated by the horizontal dashed line in A) **D** transverse section of blotch portion of the mine (location indicated by the vertical dashed line in A) **E** transverse section of intact portion of leaf lamina (indicated by left arrow in D) **F** transverse section of mined portion of leaf lamina (indicated by right arrow in D). **Ab** abaxial surface of epidermis; **Ad** adaxial surface of epidermis; **Lm** leaf mine; **Pp** palisade parenchyma; **Sp** spongy parenchyma. Scale bars =1.5, 0.15, 2.0, 3.0 mm; 500, 600 µm, respectively.

#### Etymology.

The genus name is derived from the Latin *spina* (spine) and *valva* (valve), in reference to the conspicuous spine-like process present on the male valvae. Gender feminine.

### Spinivalva gaucha

Moreira & Vargas sp. n.

urn:lsid:zoobank.org:act:BE9E4861-8769-461B-8DA0-E6BECCDD8C92

http://species-id.net/wiki/Spinivalva_gaucha

Figs 1 - 11

#### Diagnosis.

As discussed for the genus.

#### Description.

**Adult** (Fig. 1). *Head*. Frons light gray; vertex covered mostly by white scaled tuft that curves forward to the frons. Antennae mostly dark gray; scape white ventrally with pecten of light-gray hairlike scales; pedicel and flagellum ventrally whitish gray. Maxillary and labial palpi mostly white, with scattered dark-gray scales laterally. *Thorax*. Forewing mostly covered by dark-gray scales. Narrow stripe of white scales along posterior margin; a zigzag edge, formed by short, oblique white fascia, separates this stripe from the remaining, mostly dark-gray area; distal portion of apical fascia bearing brownish scales. Apical portion with transverse bar of light-gray scales that separates distally two well-defined dots, one dark gray (toward anterior margin) and one white (toward posterior margin). Fringe with scales of two sizes, mostly white at base and dark gray apically. Hindwing completely covered by dark-gray scales and with concolorous fringe. Forelegs mostly dark gray, with some white scales basally and apically on each podite, particularly on coxa. Midlegs mostly white with scattered light-brown scales, and transverse dark-gray stripes on femur, tibia, tibial spurs and tarsomeres. Hindlegs similar to midlegs, but with hair-like scales on tibia. *Abdomen*. Mostly dark gray, with transverse, V-shaped white stripes on ventral surface of segments III-VI.

**Male genitalia** (Figs 3A–D; 4A, B, D, E). As described for genus.

**Female genitalia** (Figs 3E; 4C, F). As described for genus.

#### Type material.

BRAZIL: Condomínio Alpes de São Francisco, 29°27'9.2"S, 50°37'6.6"W, São Francisco de Paula Municipality, Rio Grande do Sul State (RS), Brazil. All preserved dried and pinned, reared by the senior author from larvae and pupae collected on *Passiflora actinia* Hook. (Passifloraceae): LMCI 186, 26.V.2012, by G.R.P. Moreira, H.O. Vargas and S. Bordignon; LMCI 199, 19.XII.2012 by G.R.P. Moreira, R. Brito and F.A. Luz. HOLOTYPE: ♂ (LMCI 199-01), donated to DZUP (24.976). PARATYPES: 1♀ (LMCI 199-02), donated to DZUP (24.986); 1♂, 1♀ (LMCI 199-03 and 186-12), donated to MCNZ (81900 and 81903, respectively); 1♂, 1♀ (LMCI 199-04 and 186-15), donated to MCTP (31442 and 31443, respectively).

#### Other specimens examined.

LMCI 156: Floresta Nacional de São Francisco de Paula, 29°25'21.4"S, 50°23'26.6"W, São Francisco de Paula Municipality, RS, Brazil, collected by K.R. Barão, 13–15.V.2011, on *Passiflora actinia*. LMCI 157: Condomínio Alpes de São Francisco, 29°27'9.2"S, 50°37'6.6"W, São Francisco de Paula Municipality, RS, Brazil, collected by G.R.P. Moreira, R. Brito and G.L. Gonçalves, 28.V.2011, on *Passiflora actinia*. LMCI 164: Campus da Vale, Universidade Federal do Rio Grande do Sul (UFRGS), 30°04'12.9"S, 51°07'11.5"W, Porto Alegre Municipality, RS, Brazil, collected by R. Brito, on *Passiflora misera* Kunth and *Passiflora suberosa* L. (Passifloraceae).

LMCI 169: Centro Politécnico da Universidade Federal do Paraná, 25°26'44.1"S, 49°13'56.8"W, Curitiba Municipality, Paraná State, Brazil; 5 larvae dissected from mines collected by G.R.P. Moreira, on *Passiflora actinia*; used for DNA extraction only. Adults, dried and pinned, with the same collection data, deposited in LMCI: 4♂♂ (LMCI 156-9, 164-6, 7, 10); 1♀, (LMCI 164-9). Genitalia preparations, mounted in Canada balsam on slides, with the same collection data, deposited in LMCI: 4♂♂ (GRPM 50-11, 13, 21, 22); 4♀♀ (GRPM 50-12, 23, 32, 34). Immature stages, fixed in Dietrich’s fluid and preserved in 70% ethanol, with the same collection data series, deposited in LMCI: 2 eggs (LMCI 157-2), 4 first-instar larvae (LMCI 157-8), 5 third-instar larvae (LCMI 157-4), 6 fifth-instar larvae (LMCI 157-10), and 9 pupae (LMCI 157-5, 6). Mature leaf mines (n = 5) containing larval exuvia, mounted in glycerin on slides and stained with rose bengal, with the same collection data, deposited in LMCI, under accession numbers LMCI 186-3, 7 and LMCI 199-5, 6, 7.

#### Etymology.

The specific name is derived from the Portuguese “Gaúcho”, a term commonly used for natives of Rio Grande do Sul, the southernmost state of Brazil, where this new species was first found.

#### Immature stages.

**Egg** (Fig. 10C). Flat, ellipsoid, laid on the abaxial surface, usually close to the leaf veins; chorion translucent, larva visible through transparent area of leaf before eclosion; chorionic ultrastructure, aeropyles and micropylar area not observed.

**Larva** (Figs 5A, B, 6, 7, 8, 10F, H). Head brown, thorax and abdomen yellowish. Leaf-miner, with hypermetamorphic development and five instars, all endophyllous, prognathous and tissue feeders; that is, there is no sap-feeder instar, and all larvae have a typical spinneret and functional mandibles of the chewing type. Instars change gradually in external morphology during ontogeny, and can be identified through measurements of the head capsule, since there is no overlap between the head-capsule size of succeeding instars (Table 2). The following exponential growth equation was adjusted for the head-capsule width from larvae reared on *Passiflora actinia*: y = 0.078e^0.420x; n = 25; r = 0.99; p < 0.0001. Preliminary observations suggested that the number of instars may vary from four to five as a function of the host plant, which should be further explored.

**First instar** (Figs 5A, 6A–F). Body depressed, without setae, legs or pseudopodia. Antennae (Fig. 6C) reduced, one-segmented, nearly flush with head capsule, with four short sensilla. Stemmata absent. Labrum (Fig. 5A) moderately bilobed, with slight central notch. Mandibles (Fig. 6B) of chewing type, with three blunt teeth. Maxilla (Fig. 6E) rudimentary, uni-segmented, with two finger-like, terminal lobes. Labium relatively broad, with well-developed tubular spinneret having flared terminal opening (Fig. 6D). Hypopharynx bearing few papilliform projections basally (Fig. 6D). Labial palpi (Fig. 6F) vestigial, reduced to pair of closely appressed, slender setae.

**Third instar** (Figs 7A–F). Similar to first instar, but with body partly depressed and setae greatly reduced. Prothoracic shield slightly differentiated (Fig. 7F), nearly colorless; legs and pseudopodia absent. Antennae (Fig. 7E) bi-segmented, first segment stouter than second segment, each bearing three sensilla. Stemmata absent. Labrum (Fig. 7B) bilobed, with pronounced median notch, and several ventral, posteriorly bent papilliform projections. Mandibles of chewing type, with three teeth. Maxilla well developed, as shown in Fig. 7C. Labium broad, with spinneret similar to that of first instar, but shorter and stouter (Fig. 7C). Labial palpi (Fig. 7C) short, bi-segmented, each bearing apical sensillum. Hypopharynx with several papilliform projections basally (Fig. 7C).

**Fifth instar** (Figs 5B, 8A–L, 10F, H). Body subcylindrical, covered by microtrichia and with setae well developed; thoracic legs reduced; pseudopodia present on A3-5, A10. Head and prothoracic shield brown (Fig. 10F); remaining parts of body yellowish (Fig. 10F), changing to red before pupation (Fig. 10H). Maximum length of larvae examined 5.52 mm. Antennae (Fig. 8B) three-segmented, second segment longer than third, each bearing four sensilla. Stemmata six in number, five of them arranged close to lateral margin of head, and one inconspicuous stemma located ventrally (Fig. 8C). Labrum (Fig. 8A) bilobed, with deep median notch, similar to previous instars, but with ventral papilliform projections curved anteriorly. Mandibles similar to those of previous instars. Maxilla well developed, as shown in Fig. 8F. Labium broad, with stout, tubular spinneret having subapical opening (Figs 8G). Labial palpi (Fig. 8E) bi-segmented, basal segment longer than distal one, each bearing apical sensillum, that of distal segment longer. Hypopharynx with two sets of dense papilliform projections (Fig. 8E). Chaetotaxy: A group trisetose; L group unisetose; S group trisetose; SS group bisetose.

Thorax with prothoracic dark-brown dorsal shield well developed; one pair of legs on each thoracic segment; each leg with one pair of tarsal setae and one curved hook-like tarsal claw; one circular spiracle on each side of prothorax, near posterior margin and slightly displaced dorsally (Figs 8H–J). Protothorax chaetotaxy: D group bisetose, both located on dorsal shield; XD group bisetose, XD1 on dorsal shield and similar in length to D1 and D2; XD2 lateral to dorsal shield, about three times XD1 in length; L group bisetose, L1 dorsal to L2, slightly longer than XD2 and about three times L2 in length; SV group bisetose, posteroventral to L2, both similar to L1 in length; V group absent. Meso- and metathorax chaetotaxy: D group bisetose, length of both setae similar to prothoracic D2; D2 posterolateral to D1; L group unisetose, L1 similar to that on prothorax in length; SV group bisetose, similar to prothoracic SV group in size and position; V group absent.

Abdomen with paired, circular spiracle laterally on A1-8; prolegs on A3-5 and A10, bearing uniordinal crochets in lateral penellipse (Figs 8K–L). Chaetotaxy of A1-2, 6-7: D group bisetose, both setae very small, D1 anterolateral to D2 and posterior to spiracle; SD group unisetose, SD2 anteroventral to spiracle, length of SD2 about half of D1; L group unisetose, L1 length similar to anterior segment; SV group unisetose, length of SV1 about half of L1; V group unisetose, length of V1 similar to L1. A3-5: Similar to anterior segment, but V1 located on proleg and extremely reduced. A8: Similar to anterior segment, but SV group absent. A9: All setae lost except L group, which is similar to anterior segment. A10: D group unisetose, D2 on posterior margin; SD group unisetose, SD1 lateral to D2; L group unisetose, L1 about 1/3 length of corresponding seta on A9; SV group on proleg, bisetose; V group unisetose.

**Pupa** (Figs 5C-E, 9D–L, 10J). Maximum length of specimens examined ranged from 3.69 to 5.10 mm. General coloration yellowish, with head, thorax, and corresponding appendices darkening later in development (Fig. 10J). Vertex bearing subtriangular acute process (= cocoon cutter; Figs 9D–F) with serrated anterior edge, formed by several pointed projections that are fewer and larger at apex. Frons with 2 pairs of short frontal setae (Fig. 9D). Antennae long and slender, extending longer than pupal length; forewing reaching anterior margin of A6; proboscis extending to A2; prothoracic, mesothoracic and metathoracic legs reaching A3, A5 and A9, respectively (Figs 5C–E). Abdominal integument dorsally covered with michotrichia (Figs 9G–I). Intermediate abdominal segments with lateral margin of terga corrugated (Figs 9G–H). From A1 to A7, two micro-setae, located medially on anterior margin of terga; additional micro-setae are found laterally, located posteriorly to spiracles (Figs 9H–I). Last abdominal segment with two pairs of spines dorsally and one pair laterally, on posterior margin of tergum (Figs 9J–L).

**Host plants.** Passifloraceae: *Passiflora actinia* Hook, *Passiflora misera* Kunth and *Passiflora suberosa* L. The former, where *Spinivalva gaucha* was most frequently collected, is found primarily in forest edges in the coastal mountains of southern Brazil, where it is endemic, distributed from the Brazilian states of Espírito Santo to Rio Grande do Sul. *Passiflora suberosa* and *Passiflora misera* have broader distributions, extending to Central America, and also occur in relatively open areas occupied by shrubs and herbaceous vegetation. Details about the biology and distribution of these passion-vine species in southern Brazil were provided by Mondin et al. (2011) and Moreira et al. (2011), respectively.

**Table 2.** Variation in size among head capsules of *Spinivalva gaucha* larvae reared on *Passiflora actinia* Hook (n = 5 per instar).<br/>

**Instar**	**Head capsule width (mm)**		
	**Mean ± standard error**	**Range**	**Growth rate**
I	0.11 ± 0.002	0.10–0.12	-
II	0.19 ± 0.004	0.18–0.20	1.65
III	0.28 ± 0.009	0.26–0.32	1.52
IV	0.43 + 0.012	0.39–0.46	1.52
V	0.57 + 0.140	0.57–0.58	1.33

#### Distribution.

*Spinivalva gaucha* is known from the type locality (Condomínio Alpes de São Francisco) and the Floresta Nacional de São Francisco de Paula, both located in São Francisco de Paula Municipality, where *Passiflora actinia* plants are used as larval host plants. A few scattered specimens were also collected from an additional population located in Porto Alegre Municipality. Both municipalities are located in Rio Grande do Sul (RS), Brazil. In the Porto Alegre population, *Passiflora misera* and *Passiflora suberosa* are used as hosts. We could not find conspicuous morphological differences among the specimens collected in RS, as also corroborated by the DNA analyses. Additional *Spinivalva* specimens were collected farther north in Curitiba Municipality, Paraná, also mining *Passiflora actinia* leaves. However, as discussed below, analyses of the molecular data suggested that this population may correspond to a new species, a possibility that should be further explored. All these populations are located within the Atlantic Rain Forest domain *sensu lato* (Morellato and Haddad 2000).

#### Life history

**(**Figs 9A–C, 10A–E, G–I, K, 11). Eggs of *Spinivalva gaucha* are deposited on the abaxial leaf surface, adhered by a cement substance, close to the midrib or secondary veins (Fig. 10C). Hatching occurs through the surface of the egg adhered to the leaf; the first-instar larva moves directly into the leaf lamina, easily reaching the adaxial side of the leaf (Fig. 10D). Initially, the mine is narrow, slightly serpentine in shape, increasing in width progressively during ontogeny and becoming a blotch during the last larval instar. The larva feeds on the palisade parenchyma from the beginning to the end of the mine (Figs 10B, 11). Dark-green granular frass pellets of increasing size are found in the larva’s feeding path (Figs 10D, E), as are the head-capsule exuviae**.**

After the fifth-instar larva leaves the mine through a slit made in the blotch section (Fig. 10G) and prior to pupal moulting, it spins the pupal cocoon, usually on the adaxial leaf surface of adjacent leaves. The pupal cocoon is exophyllous, elliptical in general outline, transparent, from 7.76 to 8.74 mm long in the specimens examined. Silk filaments are woven in a tight pattern, forming a compact, flat wall that covers the pupa (Figs 9A, 10I). The cocoon periphery is adorned with several irregularly spaced, minute, light-yellow bubbles (Fig. 10I). These are not compartmentalized, showing a finely granular structured surface (Fig. 9C). They are discharged from the anus by the mature larvae to the outside through a slit made with the mandibles in the cocoon wall, which is closed soon after deposition (Fig. 9B). Throughout this process, the bubbles are manipulated by the spinneret. During adult emergence, one end of the pupal cocoon is split by the frontal process of the pupa (cocoon cutter). Generally after the adult emerges, the anterior half of the pupal exuvium (head and thorax) protrudes outside, while the posterior half remains in the pupal cocoon (Fig. 10K).

At the type locality,* S. gaucha* mines occur at low numbers on *Passiflora actinia* plants (Fig. 10A). In most cases, only one mine was found per leaf, but up to three were collected in a single leaf, and several leaves may be used per plant. There was no indication that this behavior differed from that of the other passion-vine species and populations studied. We could not find a clear pattern for the number of generations per year and the flight period.

#### Molecular phylogeny

**Figure 12.**
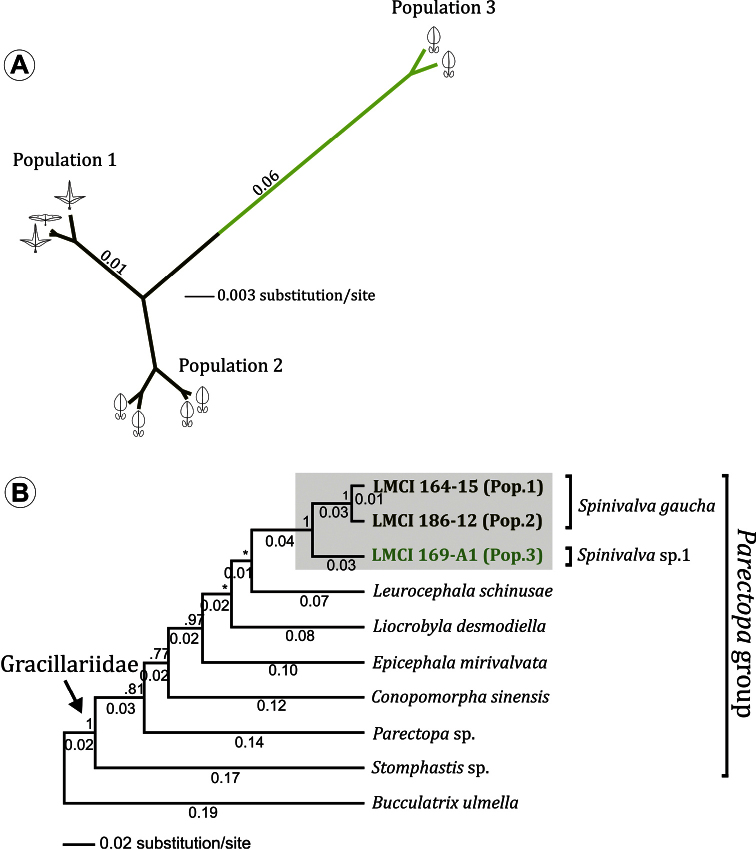
Bayesian consensus phylogeny of *Spinivalva*. **A** unrooted tree based on 1.5 Kb bp of the mitochondrial genes cytochrome oxidase *c* subunit I, tRNA-Leu and cytochrome oxidase *c* subunit II. Specimens from three different localities (termed Populations 1, 2 and 3; see Material and Methods for details), field-collected from different host plants (*Passiflora suberosa* [ ], *Passiflora misera* [] and *Passiflora actinia* []) were analyzed. Numbers indicate raw branch lengths **B** phylogenetic relationships of *Spinivalva* within the *Parectopa* group of gracillariids (sensu Kawahara et al. 2011), based on 610 bp of the barcoding region (cytochrome oxidase *c* subunit I gene). Numbers above branch indicate node support (posterior probability); those located below represent the raw branch length. A species of Bucculatricidae (*Bucculatrix ulmella*) was used to root the tree.

(Fig. 12). A total of 1583 nucleotide sites were analyzed for *Spinivalva* from different localities and host plants; 110 (7%) were variable. An unrooted Bayesian tree recovered two major groups (Fig. 12A). The first included specimens from Porto Alegre (Pop. 1), hosted on either *Passiflora suberosa* or *Passiflora misera*, together with those from São Francisco de Paula / Condomínio Alpes de São Francisco (Pop. 2) hosted on *Passiflora actinia*. The second group included individuals from Curitiba (Pop. 3) sampled on *Passiflora actinia*. The genetic divergence between these major groups was 7% (Fig. 12A). The intraspecific difference between localities in the first group was 1%. In addition, the barcode fragment analyzed recovered 610 nucleotides, including 236 (38%) variable sites. According to our phylogenetic hypothesis, *Spinivalva* was strongly supported as a monophyletic lineage within the *Parectopa* group of gracillariids (Fig. 12B). Despite the strong statistical support within this lineage of gracillariids, the internal relationships for the genera included in the *Parectopa* group were poorly resolved. *Leurocephala schinusae* and *Liocrobyla desmodiella* were placed as closest to *Spinivalva* (showing lower genetic divergence), but with weak posterior probability of node support.

## Discussion

The following characteristics suggest that *Spinivalva* gen. nov. belongs to the subfamily Gracillariinae (*sensu* Davis et al. 2011): 1) flat, scaled head; 2) maxillary palpi with four segments; 3) male abdomen bearing two coremata; 4) pupation occurring outside the mine; 5) adults resting with the anterior portion of the body inclined circa 45°. Our molecular analysis placed it within the *Parectopa* group (*sensu* Kawahara et al. 2011) in the Gracillariinae, near the genera *Leurocephala* Davis and McKay and *Liocrobyla* Meyr. From a morphological perspective, the forewing of adults of *Spinivalva* resembles those in the *Parectopa* group in general coloration, fascia arrangement, presence of apical dot, and venation pattern (Vári 1961). When compared to *Leurocephala*, a recently described genus also found in the Atlantic Rain Forest of Brazil (Davis et al. 2011), additional similarities are found in the males, in particular regarding the reduced segment VII that bears two pairs of coremata, the elongated tergum VIII, and the presence of paired gnathal lobes. However, as noted above, males of *Spinivalva* differ markedly from those of *Leurocephala* and the remaining genera of the *Parectopa* group in relation to the valva. Unlike them, it has a saccular extension with a conspicuous process bearing a stout sensillum, in association with an aedeagus that is tubular, long and slender, and a saccus with the anterior process long and tubular. These differences extend to additional lineages related to the *Parectopa* group that were not included in our molecular analysis, for example *Micrurapteryx* Spuler, 1910, *Neurobathra* Ely, 1918 (Kawahara et al. 2011), and *Chileoptilia* Vargas and Landry, 2005 (C. Lopez-Vamoonde, unpubl. data), and other genera described by Vári (1961). As far as we are aware, the existence of a saccular tubiform portion associated with the hair pencils in the coremata of *Spinivalva* has not been reported within Gracillariidae. However, detailed descriptions for coremata structures are rarely provided in the gracillariid literature, and thus one should use caution regarding the validity of this apomorphy. Bubbles adorning the pupal cocoon similar to those described here for *Spinivalva gaucha* have been found not only in other phylogenetically related genera such as *Conopomorpha* Meyrick, 1885, *Epicephala* Meyrick, 1880 and *Leurocephala* Davis & Mc Kay, 2011, but also in other lineages that are not closely related to the *Parectopa* group (*e.g*., Wagner et al. 2000, Davis et al. 2011, Hu et al. 2011). Wagner et al. (2000) speculated they provide a physical barrier, thus protecting the pupa against the attack of parasitoids and predators.

The existence of at least one “sap feeding” instar early in larval ontogeny has been considered a synapomorphy for all Gracillariidae (Kumata 1978, Davis 1987, Davis et al. 2011). However, our data showed clearly that this is not the case for *Spinivalva gaucha*, where all instars are of the “tissue feeding” type. That is, although the larvae are hypermetamorphic (early instars apodal and without stemmata, later instars with legs) as in other Gracillariidae, there is no sap-feeding in *Spinivalva gaucha*. Early-instar larvae also have mandibles of a chewing type combined with a well-developed spinneret, and with the remaining mouth parts differentiated; and after they hatch, these larvae feed on the palisade parenchyma. Palisade cells typically have well-developed, compact walls compared to those in the spongy parenchyma. The morphological characteristics in *Spinivalva gaucha* are associated with feeding on tough tissues after hatching, contrary to other gracillariid species that have sap-feeder early instars that feed by dilacerating either the leaf epidermis layers or the spongy parenchyma (*e.g*., Kumata 1978, Wagner et al. 2000, Brito et al. 2012). The absence of a sap-feeding instar was suggested for the life cycle of *Chileoptilia yaroella* Vargas & Landry, 2005, although the first instar was not described by the authors at that time. Additional studies using scanning electron microscopy recently conducted by two of us (Vargas and Moreira, unpublished data) confirmed this prediction; in this case, however, the first instar is not a leaf miner, but feeds on the tiny gynoecia within the calyx of flowers of *Acacia macracantha* Willd. (Mimosaceae) in northern Chile. These discoveries will certainly have important implications for future studies concerning the evolution of the wide diversity in feeding habits known to exist within Gracillariidae.

We found no conspicuous morphological differences at any life stage among populations of *Spinivalva* occurring in Rio Grande do Sul, independently of the host plant. These observations were corroborated by the molecular data, which showed a low divergence rate among the different populations. Consequently, we consider all these specimens to be conspecific; that is, a set of variations exists within *Spinivalva gaucha* species boundaries and among the host plants used. However, comparison of these specimens from Rio Grande do Sul with those collected from Curitiba revealed a greater divergence in mitochondrial DNA sequences. We did not study genitalia morphologies of the latter, or their immature stages, and so a decision about their taxonomic status awaits further investigation. Thus, specific diversity within the genus *Spinivalva* might be higher than described here. As discussed for the flora in general, many passion-vine species occur in the Atlantic Rain Forest, and several of them are endemic to this biome (Stehmann et al. 2009). In the future, they should be searched for the presence of this and other lineages of gracillariids. Another gracillariid species, *Phyllocnistis tethys* Moreira & Vargas, 2012, has been associated with a different passion-vine species in southern Brazil (Brito et al. 2012). However, *Phyllocnistis* larvae use a wide range of plant families as hosts (Kawahara et al. 2009). Therefore, *Spinivalva* is the first genus that is known to be particularly associated with the Passifloraceae. Passion vines are toxic to most lepidopterans, and the biological implications, if any, for such a peculiar association in herbivory also remain unknown (Brito et al. 2012).

## Supplementary Material

XML Treatment for Spinivalva

XML Treatment for Spinivalva gaucha
